# Perspectives in Brain Abscess Diagnosis and Management: A National Survey of Infectious Disease Specialists

**DOI:** 10.1093/ofid/ofaf358

**Published:** 2025-08-06

**Authors:** Ralph Habis, Melissa Canales, Susan E Beekmann, Philip M Polgreen, Kiran T Thakur, Juan Gea-Banacloche, Allan R Tunkel, Jacob Bodilsen, Arun Venkatesan

**Affiliations:** Johns Hopkins Encephalitis Center, Department of Neurology, School of Medicine, Johns Hopkins University, Baltimore, Maryland, USA; Johns Hopkins Encephalitis Center, Department of Neurology, School of Medicine, Johns Hopkins University, Baltimore, Maryland, USA; Carver College of Medicine, University of Iowa, Iowa City, Iowa, USA; Carver College of Medicine, University of Iowa, Iowa City, Iowa, USA; Department of Neurology, Columbia University Irving Medical Center, New York, New York, USA; National Institute of Allergy and Infectious Disease, National Institutes of Health, Bethesda, Maryland, USA; Department of Medicine, The Warren Alpert Medical School of Brown University, Providence, Rhode Island, USA; Department of Infectious Diseases, Aalborg University Hospital, Aalborg, Denmark; Department of Clinical Medicine, Aalborg University, Aalborg, Denmark; ESCMID Study Group of Infections of the Brain, European Society of Clinical Microbiology and Infection, Basel, Switzerland; Johns Hopkins Encephalitis Center, Department of Neurology, School of Medicine, Johns Hopkins University, Baltimore, Maryland, USA

**Keywords:** antibacterial agents, brain abscess, diagnostic modalities, neurosurgical intervention, practice patterns

## Abstract

**Background:**

Variability in causative agents, host factors, and management complexity and the lack of clear guidelines in the United States hinder the standardization of brain abscess (BA) care. This survey examines US infectious disease (ID) specialists’ perspectives on BA care, comparing practice settings and identifying key areas for future studies and guideline development.

**Methods:**

A multidisciplinary team of neurologists and ID physicians developed a 10-item online survey that was validated via a focus group and distributed to 1486 ID specialists in the Infectious Diseases Society of America's Emerging Infections Network from 7 September to 2 October 2023.

**Results:**

Of the 551 respondents (37% response rate), 116 (21%) opted out due to noninvolvement in BA cases, leaving 435 completed surveys. The most common empiric treatment (53%) consisted of third-generation cephalosporin, metronidazole, and vancomycin. Imaging at treatment completion was supported by 67%, and 75% recommended a 6- to 8-week intravenous antibiotic course. Neurosurgical intervention was primarily pursued to identify pathogens and confirm the diagnosis. Molecular diagnostics were variably used, with broad-spectrum polymerase chain reaction being the most commonly utilized test. Transitioning to oral antibiotics before 6 weeks was favored by 40%, while only 18% recommended postintravenous oral consolidation therapy. Notably, 91% endorsed the need for BA management guidelines.

**Conclusions:**

This study highlights a lack of consensus and significant variations in BA management, suggesting that current practices rely heavily on expert opinion rather than standardized protocols. These findings emphasize the need for further studies and US-specific guidelines to complement efforts of the European Society of Clinical Microbiology and Infectious Diseases and establish a more comprehensive, globally applicable standard of care.

## Teaser

This survey of 435 infectious disease physicians highlighted significant differences in brain abscess management, including varied antibiotic regimens and diagnostic approaches. Most respondents emphasized the need for formal guidelines to provide clearer recommendations on treatment duration and other management strategies.

In recent years, the incidence of brain abscesses (BAs) has been rising, as noted by nationwide studies. In Denmark, the rate increased from 0.60 to 0.90 per 100 000 person-years between 1982 and 2016, while in England, it rose from 1.24 to 2.86 per 100 000 between 1999 and 2019 [1, 2]. While this increase has been most pronounced among the elderly, age-standardized analyses suggest that demographic shifts alone do not fully explain the trend. Instead, factors such as improved access to brain imaging and the expanding use of immunomodulating treatments may be contributing to the rise [1, 2]. Advancements in diagnostic tools, such as diffusion-weighted imaging, have improved magnetic resonance imaging accuracy, with sensitivity and specificity reaching 94% and 95% in distinguishing abscesses from malignancies [3]. Additionally, molecular diagnostics have enhanced pathogen identification, while stereotactic aspiration—a minimally invasive technique—is increasingly recommended for treatment, although its superiority over open excision remains debated [4, 5].

Despite these advances, BA diagnosis and management remain complex due to the variability in pathogens and patient factors. Treatment typically involves prolonged antibiotic regimens requiring adequate central nervous system penetration and often calls for a multidisciplinary approach, including neurosurgical intervention. However, research on BA remains limited, resulting in practices frequently relying on expert opinion, with inconsistencies in care [6]. To address these gaps, the European Society of Clinical Microbiology and Infectious Diseases (ESCMID) recently published evidence-based guidelines for BA management [7]. In this survey study, we explore the perspectives of infectious disease (ID) specialists across the United States on BA diagnosis and management.

## METHODS

### Participants

The Infectious Diseases Society of America's Emerging Infections Network (EIN) is a provider-based network funded by the Centers for Disease Control and Prevention to assist public health authorities with the surveillance of emerging IDs [8]. Eligible participants for this study were physician members of the EIN with adult ID practices in North America.

### Survey Administration

The online survey was available from 7 September 2023 to 2 October 2023. Three emailed requests to answer this query were sent on 7, 14, and 21 September 2023 to 1486 ID physicians.

### Overview of the Survey

The survey was initially developed by a multidisciplinary team of neurologists and ID physicians on the basis of existing literature on BA diagnosis and management, including primary literature, reviews, and meta-analyses, with notable recent meta-analyses and guidelines [6, 9–11]. It was then piloted with a focus group of neurologists and ID physicians to optimize clarity and substance prior to finalization. Participant characteristics, including region of practice, years of experience since ID fellowship, and primary hospital type, were extracted from the EIN member database. The 10-item survey contained questions about the frequency of BA cases treated annually, empiric management, monitoring of treatment response, duration of treatment, factors in determining the need for neurosurgical interventions, use of molecular diagnostic techniques to assist in etiologic diagnosis, oral antimicrobial agents (usage, timeline, and duration), and opinions regarding development of BA guidelines (Supplementary material).

### Ethical Considerations

The EIN has an institutional review board exemption for conducting these surveys. Participation was voluntary, confidential, without financial compensation, and with no collection of patient information.

### Statistical Analysis

Categorical and qualitative variables were described by frequencies and percentages. A χ^2^ test was conducted to compare respondents and nonrespondents with respect to their years of experience. For our population of 1486 physicians, the minimum required sample size was determined per standard survey methodology. This calculation assumed a 95% confidence level, a 5% margin of error, and maximum variability (*P* = .5), followed by the application of the finite population correction [12, 13]. IBM SPSS version 29.0 was used for statistical analysis.

## RESULTS

### Survey Respondent Characteristics

Out of 1486 invited participants, 551 responded (37%), of whom 116 (21%) opted out of survey completion because they did not treat patients with BA. The final sample consisted of 435 participants who completed the survey, which exceeded the calculated minimum required sample size (n = 306). The majority (51%, n = 279) reported treating 1 to 5 patients annually. Respondents were geographically diverse, representing regions primarily across the United States. The years of practice since completing their ID fellowship also varied (Table 1). Notably, physicians who responded to the survey were significantly more likely than nonrespondents to have ≥25 years of experience after fellowship completion (*P* < .0001).

**Table 1.** Participant Characteristics

Characteristic	No. (%)
Total	435 (100)
US Census Bureau division location of practice	
New England	30 (7)
Mid-Atlantic	57 (13)
East North Central	69 (16)
West North Central	56 (13)
South Atlantic	78 (18)
East South Central	18 (4)
West South Central	30 (7)
Mountain	18 (4)
Pacific	72 (17)
Canada and Puerto Rico	7 (1)
Years’ experience since completing ID fellowship	
<5	79 (18)
5–14	164 (38)
15–24	79 (18)
≥25	113 (26)
Primary hospital type	
Community	100 (23)
Nonuniversity teaching	120 (28)
University	174 (40)
VA hospital or DOD	22 (5)
City/county	19 (4)
No. of patients with brain abscess treated per year	
<1 (not every year)	77 (18)
1–5	279 (64)
6–10	68 (16)
>10	11 (2)

Abbreviations: DOD, Department of Defense; ID, infectious disease; VA, Veterans Affairs.

### Initial Management, Monitoring, and Antibiotic Duration for BA

When respondents were presented with a clinical scenario of a 52-year-old patient with a probable BA of an unknown infection source, their willingness to delay antibiotics until neurosurgical aspiration varied. Of the respondents, 36% (n = 156) would start empiric antibiotics immediately, 34% (n = 148) would wait up to 24 hours and 19% (n = 83) up to 72 hours, and 10% (n = 45) would wait longer if symptoms did not progress (Table 2).

**Table 2.** Variations in Brain Abscess Management

Survey Item and Answer	No. (%)
How long would you hold off antibiotics before aspiration?^a^	
Start empirically right away	156 (36)
Up to 24 h	148 (34)
Up to 72 h	83 (19)
Even longer if required	45 (10)
After aspiration, what empirical antibiotics would you recommend while awaiting cultures?	
Third-generation cephalosporin + metronidazole	90 (21)
Third-generation cephalosporin + metronidazole + vancomycin	231 (53)
Fourth-generation cephalosporin + metronidazole	8 (2)
Fourth-generation cephalosporin + metronidazole + vancomycin	77 (18)
Meropenem	2 (0.5)
Meropenem + vancomycin	21 (5)
Other	6 (1)
How often should we monitor this patient with brain imaging during treatment?	
No need with clinical improvement	36 (8)
Every week	0 (0)
Every 2 wk	61 (14)
At the end of treatment only	292 (67)
Other	46 (11)
How long should the patient be treated with antibiotics postaspiration?	
4–5 wk	41 (9)
6–8 wk	325 (75)
>8 wk	22 (5)
Other	47 (11)

Responses per question: N = 435 unless indicated otherwise.

^a^n = 432.

In terms of empirical antibiotic therapy, 53% (n = 231) administered a combination of third-generation cephalosporins, metronidazole, and vancomycin; 21% (n = 90), third-generation cephalosporin and metronidazole alone; 18% (n = 77), fourth-generation cephalosporin, metronidazole, and vancomycin, and 5% (n = 21), meropenem with vancomycin (Table 2).

For monitoring during treatment, 67% (n = 292) of respondents recommended brain imaging at the end of treatment, 14% (n = 61) suggested imaging every 2 weeks, and 11% (n = 46) indicated other intervals. Only 8% (n = 35) felt that no further imaging was necessary with clinical improvement. Regarding the total duration of antibiotic treatment postaspiration, the majority (75%, n = 325) recommended 6 to 8 weeks.

### Factors Influencing the Decision for Neurosurgical Intervention and the Use of Molecular Diagnostics

**Figure 1.**
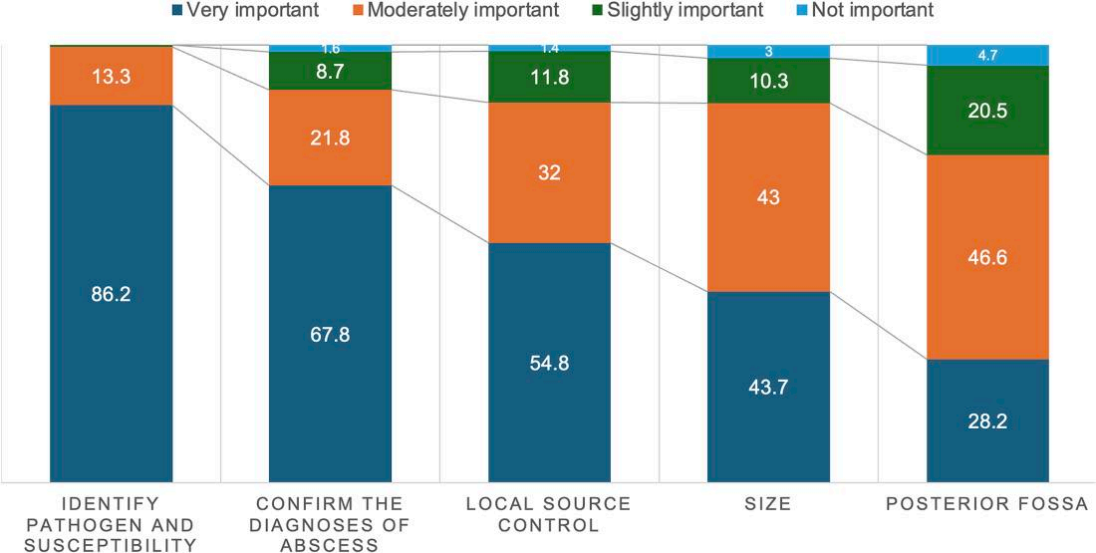
Importance of factors in determining neurosurgical intervention. This bar chart illustrates the perceived importance of various factors in the decision-making process for neurosurgical intervention in brain abscess management, based on survey responses from US infectious disease specialists. Total number of respondents: N = 435. Data are presented as percentages.

Respondents rated the importance of various factors in determining neurosurgical aspiration or excision. Identifying the pathogen and its antibiotic susceptibility was rated as the most important factor, with >86% (n = 375) of participants ranking it as very important. This was followed by confirming the diagnosis of a BA, ensuring local source control, and determining the size of the BA. The location in the posterior fossa was seen as the least important in determining neurologic intervention, with only 28% (n = 120) ranking it as very important (Figure 1).

The use of molecular diagnostics on samples from BA varied among respondents. Molecular diagnostics were unavailable for 25% (n = 109) of physicians, and 11% (n = 46) opted not to consider their use. Among the respondents utilizing molecular diagnostics, 41% (n = 179) used them in all patients with a negative culture, while 29% (n = 126) used them only in patients with a negative culture who were not responding to empiric antibiotics. Additionally, 9% (n = 37) indicated using molecular diagnostics in all patients who were immunocompromised, and only 2% (n = 8) used them in all patients.

The molecular diagnostic methods predominantly used included broad-spectrum polymerase chain reaction (PCR), as utilized by 64% (n = 277) of the respondents, and next-generation sequencing, as used by 26% (n = 79; Table 3).

**Table 3.** Use of Molecular Diagnostics and Oral Antibiotic in Brain Abscess

Survey Item and Answer	No. (%)
Do you use molecular diagnostics? Select all that apply.	
No, not available to me	109 (25)
No, never choose to use it	46 (11)
Yes, only in patients with negative culture not responding to empiric antibiotics	125 (29)
Yes, in all patients with negative culture	178 (41)
Yes, in all immunocompromised patients	37 (9)
Yes, in all patients, even those with positive culture	8 (2)
Which molecular diagnostics do you use? Select all that apply.	
Pathogen-specific PCR	32 (7)
Broad-spectrum PCR (eg, 16S rRNA)	277 (64)
Next-generation sequencing	80 (18)
Other	3 (1)
Not sure	4 (1)
Do you switch to PO antibiotics before at least 6 wk of IV treatment for typical bacterial abscess?	
Yes	174 (40)
1–2 wk	26/161 (16)
3–4 wk	62/161 (39)
>4 wk	73/161 (45)
Do you continue with PO consolidation therapy after ≥6 wk of IV antibiotic in typical bacterial brain abscess?	
Yes	78/431 (18)

Responses per question: N = 435 unless indicated otherwise.

Abbreviations: IV, intravenous; PCR, polymerase chain reaction; PO, per os (orally).

### Oral Antibiotics and Consolidation Therapy

Forty percent (n = 174) of respondents recommended transitioning to oral antibiotics before completing 6 weeks of intravenous (IV) treatment. Among these, 45% (n = 73) would transition between 4 and 6 weeks, 39% (n = 62) after 3 to 4 weeks, and 16% (n = 26) after 1 to 2 weeks. Eighteen percent (n = 78) of participants recommended continuing with oral antibiotic consolidation therapy after 6 weeks of IV antibiotics.

### The Need for Guidelines

Ninety-one percent (n = 394) of participants responded that it would be very or moderately helpful for the Infectious Diseases Society of America to develop specific guidelines for the diagnosis and management of BA. A total of 261 participants provided feedback on specific areas of BA diagnosis and management that they would like to see addressed (Table 4).

**Table 4.** Aspects of Brain Abscess Management That Respondents Would Like Addressed in Future Guidelines

Aspects	No. (%)
Diagnostic modalities	98/261 (38)
Use of molecular diagnostics	31 (12)
Indications for aspiration/biopsy	74 (28)
Other	2 (1)
Treatment modalities	220/261 (84)
Choice of empiric antibiotics	55 (21)
Choice of appropriate antibiotics	24 (9)
Duration of antibiotics	152 (58)
Timing and use of oral antibiotics	89 (34)
Frequency and role of neuroimaging in guiding management	75 (29)
Special considerations	47/261 (18)
Management of culture-negative cases	12 (5)
Pathogen-specific treatment modalities	28 (11)
Management of patients who are immunocompromised	8 (3)
Other	6 (2)

## DISCUSSION

A total of 435 ID physicians from North America participated in this survey, revealing significant variations in BA management. The most commonly used empiric therapy included a combination of third-generation cephalosporins, metronidazole, and vancomycin, with the majority recommending a 6- to 8-week IV antibiotic course followed by imaging. Pathogen identification and diagnostic confirmation were key factors influencing neurosurgical intervention, while molecular diagnostic use varied, with broad-spectrum PCR primarily employed in culture-negative cases. Notably, 91% of respondents supported the establishment of formal guidelines, with an emphasis on clarity regarding antibiotic duration and the role of oral antibiotics.

### Empiric Management of BA

While administration of antibiotics before aspiration may compromise bacterial identification, urgent initiation of empiric antibiotics may be necessary for patients who are critically ill or when aspiration is delayed. The optimal time frame for withholding antibiotics remains unclear. Recent guidelines from ESCMID conditionally recommend withholding antibiotics in nonsevere cases if aspiration occurs within 24 hours of radiologic diagnosis [7], and a Danish study showed no negative outcomes when antibiotics were delayed until neurosurgical intervention [14]. Our survey showed substantial variability: one-third followed ESCMID guidelines, one-third initiated antibiotics immediately, and one-third delayed treatment for up to 72 hours. This variability may reflect differences in patient factors or access to neurosurgical services, indicating a need for clearer evidence to assess outcomes of withholding empiric antibiotics when aspiration is delayed. Additionally, the ESCMID guidelines were made available electronically on 29 August 2023, while the survey was first distributed on 7 September 2023; this may have influenced respondents’ familiarity with the latest recommendations.

Empiric antibiotic selection for BA typically depends on infection source, local susceptibility of common pathogens, patient comorbidities, and drug pharmacodynamics. Expert recommendations commonly endorse third-generation cephalosporin and metronidazole for community-acquired BA in individuals who are immunocompetent [7, 9]. However, only one-fifth of our respondents supported this approach, with half adding vancomycin. A previous study examined the variability in broad-spectrum empiric antibiotic coverage for suspected infections across US hospitals. Many patients continue taking broad-spectrum antibiotics even when cultures return negative results [15]. These findings suggest that the empiric treatment phase, given its influence on total antibiotic use and existing variability, represents a valuable yet underutilized opportunity for antibiotic stewardship efforts in the United States.

### Monitoring and Duration of Antibiotics

Three-fourths of respondents recommended a 6- to 8-week duration for IV antibiotic therapy in conservatively managed BA, aligning with most findings in the literature [7, 9, 10]. Regarding follow-up imaging to assess treatment response, our respondents expressed a preference for imaging only at the end of the treatment period, although this is often at odds with what is reported in observational studies. Some advocate for weekly imaging for at least 2 weeks, considering a lack of improvement after 4 weeks indicative of treatment failure requiring surgical intervention [16]. Despite the significant role of neuroimaging in assessing treatment response, an observational study of 102 patients revealed that it contributes to prolonging treatment duration, emphasizing that treatment duration should not be dictated solely by imaging results [17]. Instead, clinical improvement ought to be the primary consideration, as imaging resolution frequently lags clinical progress [7, 17]. ESCMID suggests monitoring at 2-week intervals to assess treatment response, until clinical cure is evident [7].

### Factors Influencing the Decision for Neurosurgical Intervention and the Use of Molecular Diagnostics

Neurosurgical intervention in BA is indicated for diagnosis confirmation, pathogen identification, and local source control. All respondents in our survey agreed that diagnostic confirmation, pathogen identification, antibiotic susceptibility, local source control, and abscess size were important factors for neurosurgical intervention. However, despite prior studies suggesting that BA in the posterior fossa may carry increased risks of mass effect and rupture [18–20], respondents were less likely to consider this a strong determining factor for surgical intervention.

Molecular diagnostics offer significant advantages over traditional diagnostic methods, particularly in identifying a variety of bacterial taxa in a single test. This capability provides comprehensive insights into the polymicrobial nature of abscesses [21, 22]. Techniques such as next-generation sequencing are especially useful for detecting pathogens that conventional methods might miss, including a range of anaerobes from the oral cavity. These molecular-based diagnostics have identified various pathogens across different case reports, highlighting their broad applicability in clinical practice [23, 24]. When compared with traditional culture methods, molecular diagnostics can identify bacteria in 88% of culture-negative samples, significantly enhancing diagnostic accuracy [25]. In a study comparing conventional culturing with 16S rRNA amplicon sequencing in 41 BA cases, sequencing detected more bacteria, revealing a higher prevalence of polymicrobial infections missed by culture [26]. This heightened sensitivity and specificity make molecular diagnostics particularly recommended for culture-negative cases [7]. In our survey, 12% of respondents with access to molecular diagnostics reported not using it, while 45% utilized it for all culture-negative BA, with the remainder preferring to reserve its use for more challenging, refractory cases or for patients who are immunocompromised. Despite the potential benefits of next-generation sequencing in enhancing sensitivity and specificity, broad-spectrum PCR continues to be more commonly used among our respondents, likely due to greater accessibility and ease of use [27], potential cost savings, and ease of data interpretation. Molecular diagnostics are still not widely available overall, with one-fifth of respondents lacking access to them.

### Oral Antibiotics: Timing and Consolidation Therapy

Several observational studies suggest that an early transition to oral antibiotics may be safe and effective for patients with uncomplicated BA [28]. However, the evidence remains insufficient, and the ESCMID guideline has not issued clear recommendations regarding this practice. The ongoing ORAL phase 4 clinical trial aims to provide valuable insights into the efficacy of an earlier transition to oral antibiotics [29]. Forty percent of our survey participants reported switching to oral antibiotics early, despite recent German guidelines advising against this practice [30]. Similarly, a survey conducted among ID physicians across several European countries and Australia examined oral antibiotic practices and found that 50% of respondents switched to oral antibiotics before completing 4 weeks of IV treatment [11]. After a 6-week course of IV treatment, 47% of physicians from the Europe/Australia group of respondents said that they would continue with oral consolidation, while only 18% of our respondents indicated doing so [11]. ESCMID does not recommend consolidation therapy with oral antibiotics after 6 weeks of IV therapy, except in cases with specific conditions, such as permanent neuroanatomic defects or certain infections [7].

### Strength and Limitations

This study has significant strengths, including many respondents from diverse geographic regions, varying health care systems, and different levels of experience across the United States. Another advantage is the ability to compare these responses with recent guidelines and findings from a similar survey conducted in France, Sweden, Denmark, and Australia. However, the study does have limitations. Participants voluntarily joined the EIN, resulting in a nonrandom selection that may not fully represent the broader community of ID physicians in the United States. Additionally, the 37% response rate among EIN members introduces the possibility of response bias. This may be partly explained by the rarity of BA cases, which could have led some nonrespondents to consider their clinical experience too limited to justify participation. Although this rate falls below the informal benchmark of 50% to 60% often cited in physician survey research [31, 32], the final sample size (N = 435) exceeds the minimum required for statistical validity based on standard assumptions for survey precision and confidence [9, 10]. Moreover, physician surveys often yield lower response rates due to constraints on time and the specialized nature of survey topics [31]. It is also important to note that this survey captures only the opinions of ID physicians regarding the management of BA. In future surveys, it would be beneficial to gather insights from a range of specialists, such as neurosurgeons [5], to gain a more thorough understanding of the various aspects and developments in this field. As with all survey-based studies, our findings may not capture the full range of clinical nuance involved in managing BA. Differences in care may be driven by unmeasured factors, such as individual patient characteristics, institutional protocols, or regional variations in microbial epidemiology and antibiotic resistance patterns. These elements, though beyond the scope of our survey, likely play an important role in guiding decision making and may contribute to the observed variability in responses. Future studies incorporating case-specific or institutional data may help elucidate these layers of complexity.

## CONCLUSION

This survey underscores a lack of consensus in the management of BA, which varies with geographic location, highlighting that current practices are largely based on expert opinion rather than robust evidence. While recent ESCMID guidelines may be useful in assisting physicians caring for patients with BA, further research is necessary to validate these practices and develop guidelines considering specific patient populations and epidemiologic and etiologic factors.

## Supplementary Material

ofaf358_Supplementary_Data

## Supplementary Data

Supplementary materials are available at *Open Forum Infectious Diseases* online. Consisting of data provided by the authors to benefit the reader, the posted materials are not copyedited and are the sole responsibility of the authors, so questions or comments should be addressed to the corresponding author.

## Notes

***Author contributions.*** All authors contributed to the design of the study and the survey. The first draft of the manuscript was primarily written by R. H.; then, all other authors provided critical feedback and edits to the manuscript. All authors approved the final manuscript.

***Data availability.*** The data used in this study are not publicly available but could be shared on reasonable request to the corresponding author.

***Financial support.*** This work was supported by the Centers for Disease Control and Prevention (cooperative agreement 5, grant NU50CK000574).
