# Supplementary material for: Perspectives in Brain Abscess Diagnosis and Management: A National Survey of Infectious Disease Specialists
Source: Open Forum Infect Dis. 2025 Aug 6;12(8):ofaf358. doi: 10.1093/ofid/ofaf358 (PMC12341925; doi:10.1093/ofid/ofaf358)
Supplement: ofaf358_Supplementary_Data [file ofaf358_supplementary_data.pdf]

INFECTIOUS DISEASES SOCIETY OF AMERICA EMERGING INFECTIONS NETWORK QUERY:

Management of Brain Abscess

Brain abscesses are rare but serious and are associated with significant morbidity and mortality. No clinical trials on treatment have been published, nor do guidelines currently exist. Approaches to management vary considerably, including which antibiotics and for how long, as well as the timing of any switch to oral options.

We would like to learn how infectious diseases physicians manage brain abscess, including diagnostic approaches, treatment and resources they use.

Please fax to 319-384-8860 OR 319-384-7208

EMERGING INFECTIONS NETWORK QUERY
Management of Brain Abscess

Name: ______________________________

1. How many patients with brain abscess do you treat per year on average?

__ I do not see patients with brain abscess -- STOP HERE and submit
__ <1 (not every year) __ 1-5 __ 6-10 __ >10

<u>CASE</u>: A previously healthy 52y/o is admitted with headache x 4 days and new slight left-sided arm and leg weakness. MRI shows a probable right frontal lobe brain abscess of 2 cm diameter with some perifocal edema. Patient is alert, clinically stable & has normal vital signs. C-reactive protein and peripheral WBC are not elevated. The neurosurgeon plans to carry out neurosurgical aspiration.

2a. For how long would you be willing to hold off antibiotics until aspiration in this patient?

__ I would start empiric antimicrobials right away
__ Up to 24 hours as long as symptoms do not progress
__ Up to 72 hours as long as symptoms do not progress
__ Longer if required as long as symptoms do not progress

2b. The brain abscess was aspirated. Until culture results return, which empirical antibiotics would you recommend?

__ 3rd generation cephalosporin + metronidazole
__ 3rd generation cephalosporin + metronidazole + vancomycin
__ 4th generation cephalosporin + metronidazole
__ 4th generation cephalosporin + metronidazole + vancomycin
__ Meropenem
__ Meropenem + vancomycin
__ Other, *specify:*

<u>CASE UPDATE</u>: The patient improves with treatment. Brain abscess culture results grow *Streptococcus anginosus* group, *Aggregatibacter* spp., and *Fusobacterium* spp. A new MRI 2 weeks later shows slightly decreased volume of brain abscess and the patient is improving clinically.

2c. How often would you recommend monitoring this patient with brain imaging during treatment?

__ No need for further imaging with continued clinical improvement
__ Every week
__ Every 2 weeks
__ At end of treatment only
__ Other, *specify:*

2d. For how long would you treat this patient with antimicrobials (total duration after aspiration)?

__ 4-5 weeks __ 6-8 weeks __ >8 weeks __ Other, *specify:*

3. Please rate the general importance of each factor below in the decision to perform neurosurgical aspiration or excision in patients with brain abscess.

	Not important	Slightly important	Moderately important	Very important
To confirm the diagnosis of brain abscess	__	__	__	__
To identify the pathogen(s) and its antimicrobial susceptibility	__	__	__	__
To ensure local source control	__	__	__	__
Size of brain abscess	__	__	__	__
Location in the posterior fossa (assume that the abscess is accessible)	__	__	__	__

Use of Molecular Diagnostics

4. Do you use molecular diagnostics on samples of pus from patients with brain abscess?

__ No, not available to me – skip to Question 6 *[Select any that apply]*
__ No, almost never choose to use molecular diagnostics
__ Yes, only in patients with negative culture not responding to empiric antibiotics
__ Yes, in all patients with negative culture
__ Yes, in all immunocompromised patients
__ Yes, in all patients, even those with positive culture

5. If you use molecular diagnostics, which do you use? *[Select any that apply]*

__ Pathogen specific PCR
__ Broad spectrum PCR (e.g. 16s)
__ Next generation sequencing
__ Other, *specify:*
__ Not sure

Use of Oral Antimicrobials

6. Do you ever transition early to oral antimicrobials (i.e. before completion of 6 weeks or more of IV treatment) for treatment of typical bacterial brain abscess, i.e. not mycobacterial or nocardial?

__ No __ Yes, **when?** __ 1-2 weeks __ 3-4 weeks __ >4 weeks

7. Do you routinely continue treatment with oral consolidation therapy after ≥6 weeks of IV antibiotics in patients with typical bacterial brain abscess, i.e. not mycobacterial or nocardial?

__ No __ Yes

Brain Abscess Guidelines

8. How helpful would it be for the IDSA to develop and publish specific guidelines on brain abscess?

__ Not at all __ Slightly __ Moderately __ Very

9. If you would like guidelines, what specific aspects of bain abscess diagnosis and/or management would you like to see addressed?

10. Any final comments about management of brain abscess?

Thank you for completing this survey!

Please fax to 319-384-8860 OR 319-384-7208
